# Radiograph-based rheumatoid arthritis diagnosis via convolutional neural network

**DOI:** 10.1186/s12880-024-01362-w

**Published:** 2024-07-22

**Authors:** Yong Peng, Xianqian Huang, Minzhi Gan, Keyue Zhang, Yong Chen

**Affiliations:** Department of Rheumatology, Ningbo No.2 Hospital, Ningbo, Zhejiang China

**Keywords:** Convolutional neural network, Rheumatoid arthritis, Hand radiograph, Computer-aided diagnosis

## Abstract

**Objectives:**

Rheumatoid arthritis (RA) is a severe and common autoimmune disease. Conventional diagnostic methods are often subjective, error-prone, and repetitive works. There is an urgent need for a method to detect RA accurately. Therefore, this study aims to develop an automatic diagnostic system based on deep learning for recognizing and staging RA from radiographs to assist physicians in diagnosing RA quickly and accurately.

**Methods:**

We develop a CNN-based fully automated RA diagnostic model, exploring five popular CNN architectures on two clinical applications. The model is trained on a radiograph dataset containing 240 hand radiographs, of which 39 are normal and 201 are RA with five stages. For evaluation, we use 104 hand radiographs, of which 13 are normal and 91 RA with five stages.

**Results:**

The CNN model achieves good performance in RA diagnosis based on hand radiographs. For the RA recognition, all models achieve an AUC above 90% with a sensitivity over 98%. In particular, the AUC of the GoogLeNet-based model is 97.80%, and the sensitivity is 100.0%. For the RA staging, all models achieve over 77% AUC with a sensitivity over 80%. Specifically, the VGG16-based model achieves 83.36% AUC with 92.67% sensitivity.

**Conclusion:**

The presented GoogLeNet-based model and VGG16-based model have the best AUC and sensitivity for RA recognition and staging, respectively. The experimental results demonstrate the feasibility and applicability of CNN in radiograph-based RA diagnosis. Therefore, this model has important clinical significance, especially for resource-limited areas and inexperienced physicians.

## Introduction

Rheumatoid arthritis (RA) is a chronic autoimmune disease that causes swelling, pain, and stiffness in joints [1, 2]. Moreover, RA could greatly affect the health and life quality of patients, and may even lead to disability and death [3]. Meanwhile, the morbidity of RA is still relatively high, accounting for approximately 1% of the global population [4–6]. Especially in developed countries, RA affects about 5 to 50 people per 100,000 people annually [7]. However, the etiology of RA remains unclear, and there is still no cure for RA. The treatment of RA focuses on preventing it from progressing to permanent damage by alleviating pain and reducing inflammation [8, 9]. Therefore, it is crucial to intervene and control RA through timely and accurate diagnosis, especially early diagnosis, to prevent it from being a permanent disease.

At present, the examination and diagnosis methods of RA mainly include laboratory examination, imaging examination, arthroscopy, and arthrocentesis [5]. In particular, the radiograph is one of the most common and primary methods of RA diagnosis because of its speed, affordability, and effectiveness in visualizing lesions. However, it is found in practice that physicians may misinterpret radiographs, which may be contributed to several reasons, such as inexperience, poor image quality, tiredness caused by long-term reading, and their own subjectivity. Meanwhile, it is easy to dismiss the ambiguous lesion characteristics, especially tiny lesions and early RA, leading to false negative results and mistaken diagnoses. Especially in resource-limited areas, the misdiagnosis rate of RA is higher due to outdated detection equipment or insufficient experience of physicians. To overcome these challenges and reduce misdiagnosis rate, researchers have begun to develop computer-aided diagnosis (CAD) systems to assist doctors in obtaining more accurate results. CAD systems are capable of detecting lesions and graphically displaying the diagnostic results to physicians. CAD systems can locate, diagnose and quantitatively analyze the lesions on medical images, thereby reducing the misdiagnosis and missed diagnosis of lesions by physicians, and improving diagnostic accuracy and rate. Therefore, it is clinically significant to build a CAD system to effectively assist physicians in completing RA diagnosis accurately and efficiently, especially for resource-limited areas and physicians with insufficient experience.

In recent years, deep learning has developed rapidly and has been widely used in various computer vision tasks [10–12]. In particular, convolutional neural network (CNN) is one of the most popular and universal architectures in deep learning [13–15]. Furthermore, CNN is also well applied in the field of medical image analysis, such as nodule and tumor detection [16–18], organ segmentation [19–21], and cancer screening [22, 23]. CNN-based CAD systems are characterized by objectivity, efficiency, and high sensitivity [24]. Therefore, we consider CNN to be a natural candidate for automatic radiograph-based RA diagnosis to improve the diagnostic accuracy and efficiency of physicians.

In this study, we aim to explore the feasibility and applicability of various CNN architectures in radiograph-based RA diagnosis to assist physicians in diagnosing RA accurately and efficiently. For this purpose, we propose a CNN-based model for RA recognition and staging. Specifically, the model is designed based on five popular and universal CNN architectures, such as AlexNet [13] and VGG16 [25]. The main contribution of this study can be summarized as follows: 1) We present a CNN-based automatic diagnostic model to assist physicians in diagnosing RA accurately and quickly. Therefore, physicians can quickly know whether a patient suffer from RA and the stage of RA by simply feeding the hand radiographs into the model. 2) To improve the reliability and interpretability of the presented model, we use t-SNE technology to visualize the representations to display whether the samples of same category can be effectively clustered into the same cluster. 3) We analyze and compare five different CNN architectures in two tasks. Thus, we determine the best model structure in RA recognition and RA staging to assist physicians in diagnosing RA.

**Fig. 1:**
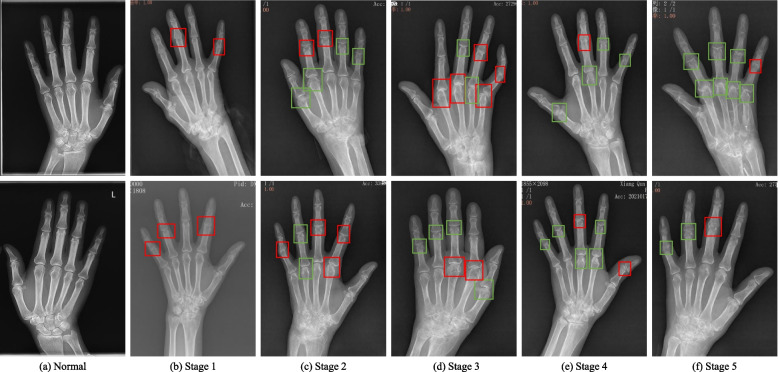
Hand radiographs with RA stage annotations. **a**-**f** represent the normal patients and patients with RA at different stages. The top and bottom rows represent the right and left hands, respectively. For the same patient, the red box represents the highest RA stage and the green box represents other stages. For example, for a RA (stage 3) patient, the red box represents stage 3, while the green box represents stage 1 or 2

## Materials and methods

### Image acquisition

The hand radiographs of RA are mainly collected from two general hospitals in Ningbo, China from January 2020 to March 2023. All protected patient health information contained in the DICOM header is eliminated by data masking approaches, including patient name, institution ID, and referring physician name. RA stages may vary in different hands of the same patient due to work and lifestyle factors. Therefore, to make the results more accurate, we separate all radiographs containing both hands into two radiographs, with only the left or right hand included in each radiograph. We finally collect 344 hand radiographs. The study is approved by the Ethics Committee of Ningbo No.2 Hospital.

### Image annotation

We divide the patients into normal and RA with five stages, according to medical guidelines [7, 26] and the actual requirements of the hospital. Meanwhile, if a hand suffers from RA in multiple joints with different stages, we consider the most severe RA to be the final stage of the hand. To annotate the RA stages as accurately as possible, we employ a two-stage procedure for interpreting radiographs. In the first phase, two physicians annotate the radiographs separately according to the annotation scheme. The purpose of the second phase is to calibrate the annotations in the first phase. If there are discrepancies in the annotations between the two physicians in the first stage, they will discuss to determine the final annotation. We illustrate the location and stage of the RA lesion on the hand radiograph in Fig. 1.

### Data pre-processing

We randomly divide the RA dataset into a training set and a test set at a ratio of 7 : 3, as shown in Table 1. Meanwhile, to prevent potential data leakage, both the left and right hands of the same patient are only in the same dataset. Eventually, 240 radiographs are used to train the model, and 104 radiographs are used to evaluate the model. Due to the different resolutions of the original radiographs, we resize each radiograph to $224 \times 224$ pixels to maintain the sample consistency while training the model. Furthermore, the appearance of radiographs, such as brightness and contrast, varies widely due to the acquisition sources and radiation dose. Therefore, we normalize each radiograph to scale the pixel intensity into the range of [0, 255] .

### Data augmentation

Robust deep learning models need to be trained with large amounts of samples. However, high-quality annotated medical images are scarce due to the high cost of annotation. Therefore, we implement an implicit expansion of samples by applying data augmentation techniques to prevent CNN from learning irrelevant patterns and over-fitting [27]. These data augmentation approaches include random rotation, translation, and horizontal and vertical flipping.

**Table 1:** Description of training and test datasets

Class	Training	Test	All
**Normal**	26	13	39
**Stage 1**	33	14	47
**Stage 2**	54	22	76
**Stage 3**	56	24	80
**Stage 4**	38	17	55
**Stage 5**	33	14	47
**Total**	240	104	344

### Model training

Five different popular CNN architectures are used to build the RA diagnostic model, including AlexNet [13], VGG [25], GoogLeNet [28], ResNet [14], EfficientNet [29]. For fair comparison, we optimize all five architectures using the same parameters. Here, we train the model using the AdamW optimizer with a batch size of 64. Meanwhile, the initial learning rate and weight decay are set to 1e-5 and 1e-2, respectively. All models are trained for 100 epochs. In particular, since there are many variants of VGG, ResNet, and EfficientNet, we only train VGG16, ResNet50, and EfficientNetB2, which are the most commonly used of these networks. Furthermore, all networks are implemented by PyTorch, and all experiments are performed on two NVIDIA RTX 2080Ti GPUs with 11GB of memory. The details of these CNN architectures are as follows:

AlexNet: AlexNet is a CNN architecture designed for image classification. It consists of five convolutional layers, some followed by max-pooling layers, and three fully connected layers. Especially, it introduces the ReLU activation function and GPUs to improve training speed and employed dropout to reduce over-fitting. It is also using data augmentation techniques to accelerate convergence.

VGG16: VGG16 consists of 16 layers, including 13 convolutional layers with $3 \times 3$ filters and 3 fully connected layers. The convolutional layers are stacked on top of each other to increase the depth of the feature map while maintaining the spatial resolution by maximizing the pooling layer. It also employs the ReLU activation function and uses a softmax classifier in the last layer. The architecture achieves high accuracy in image classification on the ImageNet dataset.

GoogLeNet: GoogLeNet is a type of CNN based on the Inception module [28] designed for efficient computation and high accuracy. The Inception modules use multiple filter sizes ($1 \times 1$, $3 \times 3$, $5 \times 5$) and pooling operations within the same layer to capture different spatial features. The network consists of 22 layers, including 9 Inception modules, and employs global average pooling at the end instead of a fully connected layer to reduce parameters and prevent over-fitting. GoogLeNet demonstrates the effectiveness of multi-scale feature extraction.

ResNet50: ResNet50 is one of the most commonly used CNN architectures. It contains 50 layers and is designed to address the problem of gradient vanishing by utilizing the residual learning. The model consists of multiple residual blocks, each containing a convolutional layer, batch normalization, and ReLU activation function. The residual blocks allow the network to learn identity mapping, which makes it easier to train deeper model. It improves the training efficiency and accuracy of deep networks and greatly advances the development of deep learning.

EfficientNetB2: EfficientNetB2 employs a technique of compound model scaling to scale the depth, width, and resolution, aiming to balance the performance and efficiency. It consists of multiple mobile inverted bottleneck convolution (MBConv) blocks and squeeze-and-excitation (SE) optimization, which enhances the feature extraction capability. Comparing with traditional CNN architectures, it also employs SiLU (Swish-1) activation function and batch normalization techniques to achieve superior performance in image classification tasks with fewer parameters and lower computational cost.

### Evaluation metrics

We use the receiver operating characteristic (ROC) curve to show the performance of a classification model at all classification thresholds. The ROC curve is obtained by plotting the true positive rate against the false positive rate at different threshold settings. We define the area under the ROC curve (AUC), accuracy, sensitivity, specificity, and f1 to evaluate the model [30]. AUC measures the entire area underneath the entire ROC curve. The other metrics are defined as follows.

1 $$\begin{aligned} Accuracy = \frac{TP + TN}{TP + TN + FP + FN} , \end{aligned}$$

2 $$\begin{aligned} Sensitivity = \frac{TP}{TP + FN} , \end{aligned}$$

3 $$\begin{aligned} Specificity = \frac{TN}{TN + FP} , \end{aligned}$$

4 $$\begin{aligned} F1 = \frac{2 * TP}{2 * TP + FP + FN} , \end{aligned}$$

True positive (TP) means that the RA sample is correctly classified. True negative (TN) means that the normal sample is correctly classified. False positive (FP) means that the normal sample is misclassified as RA. False negative (FN) means that the RA sample is misclassified as a normal sample.

## Results

### RA recognition and staging

**Fig. 2:**
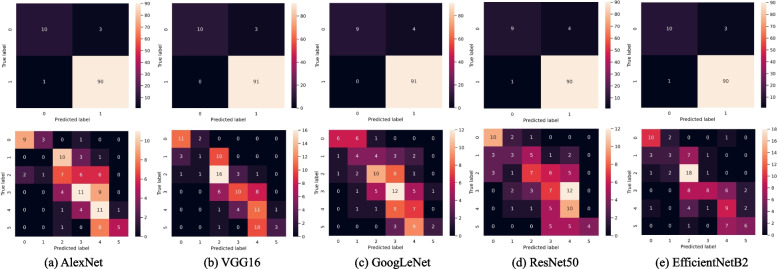
Confusion matrix. **a**-**f** represent the different CNN architectures. The top and bottom rows represent the confusion matrix of RA recognition and staging obtained by different CNN architectures, respectively. For each confusion matrix, the horizontal axis represents the model predicted label, and the vertical axis represents the true label

We evaluate the CNN-based model in RA recognition and staging tasks, as shown in Tables 2 and 3. The experimental results show that the model achieves excellent AUC with high sensitivity in both tasks. Meanwhile, we display the confusion matrix to efficiently visualize misclassified samples, as shown in Fig. 2.

**Table 2:** Comparison of different methods on RA recognition task (%)

Method	AUC	Accuracy	Sensitivity	Specificity	F1
AlexNet	95.35	96.15	98.90	76.92	97.83
VGG16	90.03	97.12	100.0	76.92	98.38
GoogLeNet	97.80	96.15	100.0	69.23	97.85
ResNet50	93.03	95.19	98.90	69.23	97.30
EfficientNetB2	95.77	96.15	98.90	76.92	97.83

**Table 3:** Comparison of different methods on RA staging task (%)

Method	AUC	Accuracy	Sensitivity	Specificity	F1
AlexNet	77.36	53.04	89.54	44.27	40.84
VGG16	83.36	56.57	92.67	47.72	44.71
GoogLeNet	80.32	66.19	84.45	61.24	48.35
ResNet50	78.87	63.30	82.38	58.55	46.32
EfficientNetB2	83.06	69.39	81.45	66.36	47.36

For RA recognition, all methods achieve an AUC greater than 90% with high sensitivity (over 98%). Meanwhile, the accuracies of the model based on AlexNet, VGG16, GoogLeNet, ResNet50, and EfficientNetB2 are 96.15%, 97.12%, 96.15%, 95.19%, and 96.15%, respectively. Confusion matrix also show that RA samples are rarely or even not classified as normal samples.

For RA staging, all methods can also achieve an AUC over 77% with high sensitivity (over 81%). Although the accuracy of all methods is not very high (below 70%), we can find from the confusion matrix that most of the prediction errors occur between adjacent RA stages. Meanwhile, RA samples are rarely predicted as normal samples. Therefore, the model achieves high sensitivity with low false negatives.

**Fig. 3:**
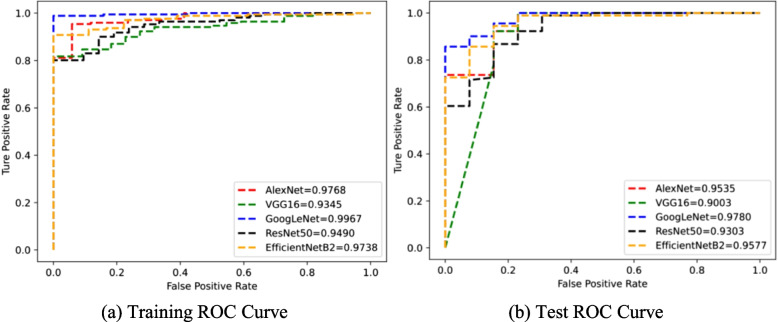
The ROC curve on RA recognition. **a** and **b** represents the ROC curve of training set and test set

**Fig. 4:**
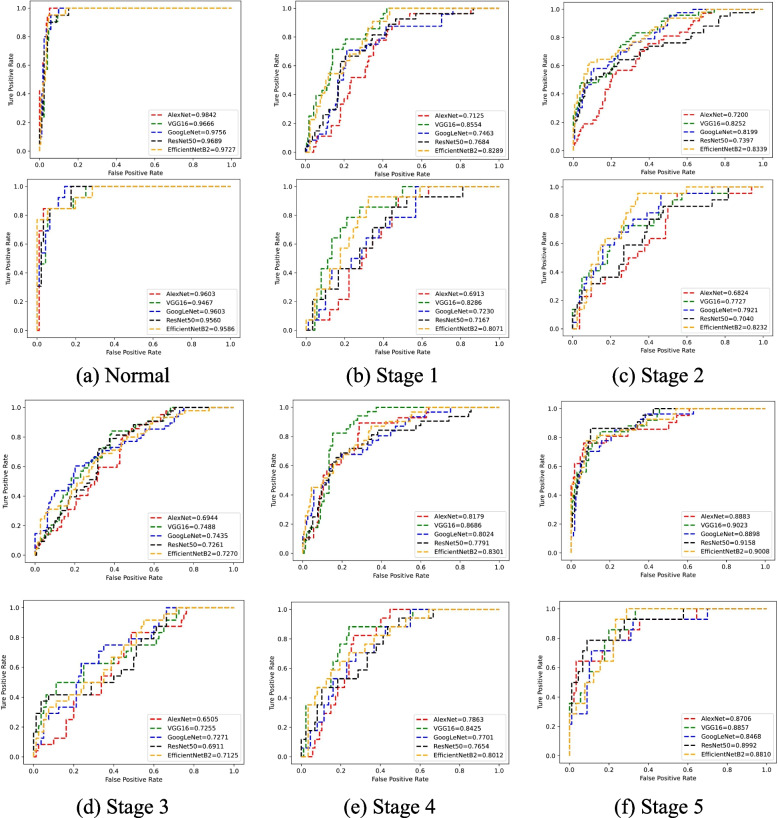
The ROC curve on RA staging. **a**-**f** represent the normal patients and patients with RA at different stages. For each RA stage, the top and bottom figures represent the training ROC curve and the test ROC curve, respectively

### ROC curve

The ROC curve is a relatively stable metric for selecting a potentially optimal model, especially for unbalanced samples. As shown in Figs. 3 and 4, the difference between the AUC obtained by all methods on the corresponding training and testing is no more than 6%, which proves that our proposed model is not over-fitting. Especially for RA recognition, all AUCs are more than 90.0% in the case of unbalanced samples, and the ratio of RA to normal samples is about 7 : 1. For RA staging, the model has a relatively higher AUC for predicting early-stage (including normal) or late-stage RA than predicting mid-stage RA. That is because the characteristics between different mid-stage RA are not very distinct.

**Fig. 5:**
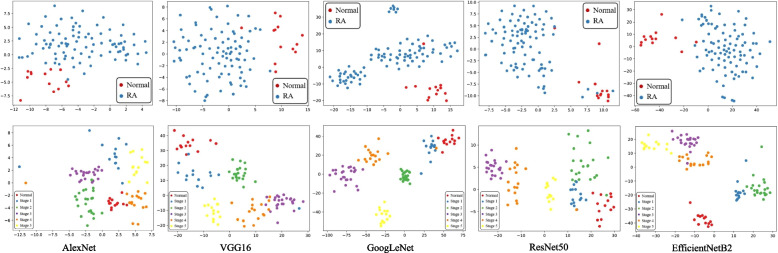
Representation visualization by t-SNE. The columns represent the different CNN architectures. The top and bottom rows represent the RA recognition and staging, respectively. In each figure, different colors represent the representations of different classes of RA samples

### Visualization

The predictive power of a model is attributed to its ability to learn discriminative representations. For the purpose of improving visual interpretation, we visualize discriminative and aggregated representations of input images by t-SNE visualization [31]. In Fig. 5, different colors represent different classes of RA samples. We can observe three different aspects. First, samples with the same class are clustered together. Second, samples from different classes are separated. Third, RA samples are relatively scattered in the RA recognition task due to the significant variations between different RA stages.

**Fig. 6:**
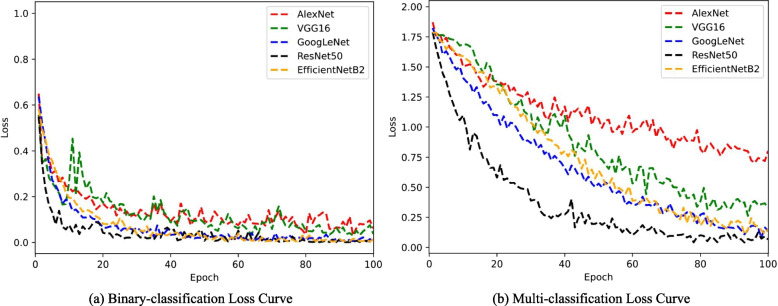
Comparing loss functions on the training dataset. **a** and **b** represent the RA recognition and staging loss, respectively

### Loss curve

Figure 6 shows the variation of the loss function for different methods in RA recognition and staging, respectively. It is clear that the loss decreases as the number of epochs increases during training. For RA recognition, the training loss of ResNet50 seems to reach the threshold within 10 epochs, while other models basically converge within 40 epochs. However, the training loss for RA staging converges much slower than that for RA recognition. For RA staging, ResNet50 converges around epoch 60, while other models converge around epoch 90. The convergence of loss proves the stability and generalization of the proposed model.

## Discussion

In this study, we present a fully automated diagnostic model based on CNN architectures, aiming to assist physicians in diagnosing RA. We demonstrate the feasibility and effectiveness of the model by analyzing and comparing five well-known CNN architectures. The model achieves excellent AUC and high sensitivity in both RA recognition and staging. In the test set, GoogLeNet achieves AUC of 97.80% and sensitivity of 100.0% in RA recognition, and VGG16 achieve AUC of 83.36% and sensitivity of 92.67% in RA staging. The experimental results demonstrate that the model can diagnose RA quickly and accurately. Therefore, the model can effectively assist physicians in diagnosing RA quickly and accurately to minimize misdiagnosis rates, especially for physicians working in resource-limited areas and lacking experience.

Many researches [32, 33] treat deep learning model as a black box as it lacks interpretability, which limits its application in the field of medical image analysis. In our study, we adopt the t-SNE-based visualization technique to improve the model interpretability. In the RA recognition and staging tasks, t-SNE visualization clearly shows which samples the model maps to the same clusters. From Fig. 5, it can be observed that although some sample points fall into clusters, this mainly occurs among samples from adjacent stages. The reason is that for samples from adjacent stages, the differences in lesions are not distinct. However, for the majority of samples and those with larger differences between stages, the representation clusters exhibit clear distinctiveness.

To our knowledge, there are only a few previous studies that have focused on the diagnostic performance of CNN-based approaches to diagnose RA within the hand joints on radiographs [26, 34–36]. Morita et al. [34] propose the finger joint detection method estimation method using support vector machine on 45 RA radiographs. Experimental results show that the proposed method detects finger joints with an accuracy of 81.4%, and estimated the erosion and joint space narrowing score with an accuracy of 50.9% and 64.3%, respectively. They use a small number of images for training and testing, which may lead to over-fitting. Ureten et al. [35] develop an automated diagnostic method using a CNN on hand radiographs to help physicians diagnose RA. The method is trained on 135 right-hand radiographs and tested on 45 radiographs with a sensitivity of 68.18% and a specificity of 78.26%. Compared with our method, they achieve a lower sensitivity. To identify RA from normal patients, Mate et al. [36] also propose a CNN-based classification model and evaluate the model on a dataset containing 290 radiographs. The results show that the method achieves an accuracy of 94.46%, as well as a sensitivity of 95.0% and a specificity of 82.0%. However, the above two studies focused only on RA recognition without staging, yet early-stage RA is crucial for RA treatment and rehabilitation. Hioki et al. [26] develop an automatic assessment system for RA based on deep learning, which simultaneously realizes the RA recognition and staging. However, the model is validated on only 50 radiographs, which may lead to over-fitting and weak robustness.

In conclusion, although existing studies have shown relatively high performance in RA diagnosis, these studies have two common shortcomings, namely small samples or limited tasks. The former tends to lead to over-fitting, which impairs the generalization and robustness of the model [26, 34, 35]. To address the issue, we collect and annotate a moderate number of samples, as well as these samples are implicitly expanded by data augmentation techniques when training the model. The latter neither fully exploits the potential of the model nor satisfies the clinical needs, resulting in weak feasibility and applicability [35, 36]. Thus, we validate five popular CNN architectures on two RA diagnosis tasks to exploit the potential of the presented model. The experimental results show the clinical reliability and significance of the model.

Furthermore, it is well known that the strong classification ability of CNN benefits from its discriminative representational learning. Thus, the discriminative power of our proposed model is mainly attributed to the following two aspects. On the one hand, the model learns aggregated representations within the same category and separated representations among different classes. Particularly, the model is still able to learn discriminative representations under the sample imbalance. On the other hand, the model has a low false negative rate for RA recognition, i.e., few or even no RA samples are classified as normal. Meanwhile, for RA staging, most prediction errors also occur between adjacent RA classes. This is because there may be more than one joint with RA in the same hand with different stages, and we take the highest stage as the final RA label for the hand. In addition, joints in adjacent RA stages are relatively similar in shape and structure. Both aspects increase the difficulty of representational learning. Nevertheless, the results demonstrate the effectiveness of the CNN-based model for RA diagnosis.

Accurate diagnosis of RA patients has important clinical significance for the treatment of RA. There is a lack of reliable CAD systems for RA diagnosis. In this study, we demonstrate the feasibility and applicability of the CNN-based model in RA diagnosis. Furthermore, The potential application of the model is that it can help in identifying patients with early RA. Since there is currently no cure for RA, it is crucial to control it at an early stage.

Although the proposed CNN-based model achieves excellent performance in RA recognition and staging, there are still the following limitations or challenges. Firstly, there is a relatively low accuracy of the RA staging due to the small differences between adjacent RA stages. Therefore, it is necessary for future studies to improve the learning ability of discriminative representations based on deep learning methods such as contrastive learning. Secondly, there is a challenge of insufficient high-quality annotated images in medical image analysis. We believe that transfer learning or unsupervised learning will be an effective way to solve the problem. Finally, RA examinations include radiographs, laboratory examinations, and others. It is a highlight of future research to build multi-modal learning methods that integrate different data for more accurate diagnosis.

## Conclusion

In this study, we present an innovative CNN-based approach to build an automatic RA diagnostic model, aiming to assisting physicians in diagnosing RA quickly and accurately. For this purpose, we explore the feasibility and applicability of five different popular CNN architectures based on radiographs. In particular, GoogLeNet and VGG16 achieve the best results in RA recognition and staging with AUCs of 97.80% and 83.36%, respectively. Extensive experimental results demonstrate that the presented CNN-based model achieves excellent performance in both tasks. Overall, the model can assist physicians in diagnosing RA, especially in resource-limited areas and for inexperienced physicians. In future work, we will continue to optimize the network and incorporate patients’ laboratory data and multi-center data into the model to build a more comprehensive diagnostic model.

## Data Availability

The data that support the findings of this study are available on reasonable request from the author(E -mail: nbdeyycy@163.com). The imaging data were not publicly available because of restrictions (containing information that could compromise the privacy of research participants).
